# Capsule Endoscopy in the Small Bowel Crohn's Disease

**DOI:** 10.1155/2014/529136

**Published:** 2014-03-11

**Authors:** Federico Argüelles-Arias, Juan Rodríguez-Oballe, Calixto Duarte-Chang, Luisa Castro-Laria, Josefa María García-Montes, Ángel Caunedo-Álvarez, Juan Manuel Herrerías-Gutiérrez

**Affiliations:** Clinical Unit of Gastroenterology, University Hospital Virgen Macarena, Seville, Spain

## Abstract

CD is a chronic inflammatory disorder associated to mucosal and transmural inflammation of the bowel wall. It is well known that CD can affect the entire gastrointestinal. Therefore, ileocolonoscopy and biopsies of the terminal ileum as well as of each colonic segment to look for microscopic evidence of CD are the first-line procedures to establish the diagnosis. However, it has been observed that up to 30% of the patients have only small bowel involvement. Evaluation of the small bowel has been made with radiological procedures, barium radiography, and abdominal computed tomography or by ileocolonoscopy or enteroscopy, but they have many recognized limitations. CE is undoubtedly a very useful diagnostic tool proposed to observe small-bowel lesions undetectable by conventional endoscopy or radiologic studies. We review different studies that have been published reporting the use of CE in suspected and evaluation of the extension or the recurrence in CD and also its use in pediatric population and its complications.

## 1. Introduction

The incidence and prevalence of Inflammatory Bowel Disease (IBD) have increased in the past 50 years, specifically up to 6–15/100,000 and 50–200/100,000 persons, respectively, for Crohn's disease (CD) in the western countries [1]. These patients may have unexplained fever, weight loss, anaemia, pain, or diarrhea, but depending on the digestive segment affected the symptoms will be different. Although there is no gold standard test for the diagnosis of small bowel CD [2], diagnosis should be made using a combination of clinical, endoscopic, radiological, histology, and biochemical tests.

CD is a chronic inflammatory disorder associated with mucosal and transmural inflammation of the bowel wall. It is well known that CD can affect the entire gastrointestinal tract from the mouth to the anus although the most common presentation is ileum-colon, 50% of the cases [1]. Therefore, ileocolonoscopy and biopsies of the terminal ileum as well as of each colonic segment to look for microscopic evidence of CD are the first-line procedures to establish the diagnosis [3]. However, it has been observed that up to 30% of the patients have only small bowel involvement [4, 5]. Jejunal lesions are also detected in more than half of the patients with CD and the prevalence of jejunal lesions is higher when the terminal ileum is involved [6]. It is well known that the presence of jejunal lesions is associated with an increased risk of further clinical relapse [7] and therefore an early and rapid diagnosis is necessary.

Evaluation of the small bowel has been made with radiological procedures, barium radiography, and abdominal computed tomography (CT) or by ileocolonoscopy or enteroscopy, but they have many recognized limitations. In this context, Magnetic Resonance Enterography (MRE) has emerged as a modern technique with increasing implantation and is recognized as a useful and effective tool for the diagnosis of intestinal injury. However, it is not efficient to display subtle or mucosal lesions and versus the capsule endoscopy (CE) it has shown lower sensitivity for the diagnosis of small bowel CD [8]. One study [9] compared CE with MRE in patients with suspected small bowel disease. CE depicted a higher number of inflammatory lesions in the jejunum and proximal ileum compared with MRE. CE is undoubtedly a very useful diagnostic tool proposed to observe small bowel lesions undetectable by conventional endoscopy or radiologic studies.

## 2. Capsule Endoscopy in Suspected and Established Crohn's Disease

Many studies have reported the use of CE in suspected CD with previous negative ileocolonoscopy. Herrerías et al. [10] studied 21 patients who underwent CE because of abdominal pain, diarrhea, weight loss, fever, anemia, and elevated C-reactive protein. Colonoscopy and small bowel series were negative in all patients. In nine patients (43%) the CE found lesions compatible with CD. Fireman et al. [11] also reported a 71% yield, in which 12 of 17 patients with normal small bowel series and colonoscopy, but with a high clinical suspicion of having CD, were found to have lesions compatible with this condition: aphthae, linear or irregular ulcers, and mucosal fissures. In other studies, the CE diagnosed Crohn's disease in 26% [12], 59% [13], or 52,4% [14] of the patients. As we can see, results are heterogeneous and depend on study definitions, design, and follow-up.

The improvement of CE for the study of small bowel has happened because of the inconsistent results of the radiological studies. Small Bowel Follow-Through (SBFT) and Enteroclysis are limited by poor sensitivity for early or subtle inflammatory lesions of CD and for ionizing radiation exposure; many studies have shown better yield for CE [15–20]. Cross-sectional imaging techniques such as CT Enterography (CTE) and Magnetic Resonance Enterography (MRE) have replaced the traditional techniques with better results (see Table 1). The largest comparative study of multiple small bowel imaging modalities involved a comparison of CE, CTE, and MRE performed after ileocolonoscopy [21]. The results reported a significantly superior detection of CD in the proximal small bowel by CE compared with both CTE and MRE. In suspected or newly diagnosed CD, MRE and CTE have comparable sensitivities and specificities and, in patients without endoscopic or clinical suspicion of stenosis, CE should be the first-line modality for detection of small bowel Crohn's disease beyond the reach of the colonoscope. Overall, these comparative studies suggest that CE is more sensitive than SBFT and may be more sensitive than cross-sectional imaging.

Subsequently, two meta-analyses that study the efficacy of the CE have been published. Triester et al. [22] published the first one, including nine studies with 250 patients comparing CE with other imaging techniques of the small bowel, concluding that CE is superior to all other modalities for diagnosing nonstricturing small bowel CD, with a number needed to test (NNT) of 3 to yield one additional diagnosis of CD over small bowel barium radiography and NNT = 7 over colonoscopy with ileoscopy. The other one has recently been published [23] and a total of 12 trials were compared. Eight trials (*n* = 236) compared CE with colonoscopy plus ileoscopy, four trials (*n* = 119) compared CE with Computerized Tomography Enteroclysis (CTE), two trials (*n* = 102) compared CE with Push enteroscopy, and four trials (*n* = 123) compared CE with MRE. Again this meta-analysis has demonstrated that CE is superior to small bowel radiography, CTE, and colonoscopy plus ileoscopy in the evaluation of suspected CD patients.

Furthermore, the yield of the CE has been compared with push enteroscopy and double-balloon enteroscopy (DBE). CE has a higher yield than push enteroscopy [20] and recently two meta-analyses have been published, concluding that CE and DBE have comparable diagnostic yield in small bowel diseases [24, 25]. Consequently, CE should be the initial diagnostic test and, because of its therapeutic capabilities, DBE may be indicated in patients with a positive finding on CE requiring a biopsy or therapeutic intervention.

Also, in order to determine patient burden and patient preference for MRE, CE, and balloon-assisted enteroscopy in patients with suspected or known CD or occult gastrointestinal bleeding, a study was developed and CE was preferred to MRE and balloon-assisted enteroscopy and it also had the lowest burden [26].

**Figure 1:**
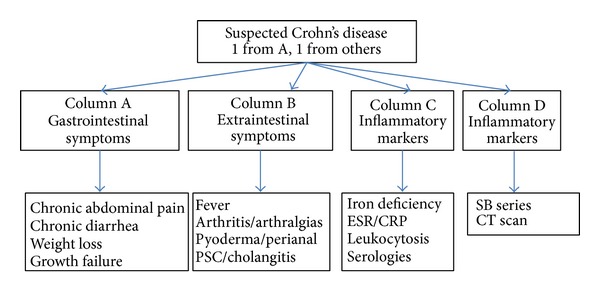
Criteria for suspected Crohn's disease [30]. PSC: primary sclerosing cholangitis; ESR: erythrocyte sedimentation rate; CRP: C-reactive protein; SB series: small bowel series; CT scan: computed tomography scan.

It is important to take into account that many lesions described in studies of suspected CD are not specific and this could explain the variability of the “diagnostic yield” of CE. Also, it must be considered that the “diagnostic yield” is different to either “sensitivity” or “specificity” [27]. Yield of CE varies depending on the type of patient, and so forth, and it is lower when performed in patients with only abdominal pain [28] and in patients with abdominal pain and diarrhea [29]. In this way, the first consensus of the International Conference on Capsule Endoscopy (ICCE) concluded that CE is capable of identifying lesions of the mucosa of the small bowel overlooked with other imaging techniques and also defines CD suspicion group [30]. Further on, in order to improve the diagnosis yield of the CE, the ICCE proposed an algorithm including the main suspicion criteria for CD [31] (Figure 1). Colonoscopy with ileoscopy must always be performed prior to capsule endoscopy, considering it for CD diagnosis or exclusion if the patient presents suspicious symptoms (abdominal pain or persistent diarrhea) as well as extraintestinal manifestations, alteration of inflammatory markers, or abnormalities in other imaging tests [32]. In this sense, fecal calprotectin in small bowel CD can play an important role. Fecal calprotectin is a noninvasive marker of gastrointestinal inflammation with advocated diagnostic precision to differentiate Inflammatory Bowel Disease from non-IBD diagnoses. A recently published article has assessed the sensitivity and specificity of fecal calprotectin in suspected CD [33]. With a 50 mg/kg cut-off, CD in the small intestine and colon was diagnosed with 92% and 94% sensitivity, respectively, and the overall sensitivity and specificity were 95% and 56%. Therefore measurement of fecal calprotectin levels in patients with CD suspicion prior to referral for CE is a useful tool. In another study, nevertheless, the cut-off was higher and a fecal calprotectin >200 *μ*g/g was associated with higher CE yield (65%) and confirmed CD in 50% of cases; when fecal calprotectin <100 *μ*g/g (NPV 1.0), CE is not indicated [34]. In any case, more studies must be done because in another paper fecal biomarkers calprotectin and S100A12 have moderate specificity but low sensitivity [35].

The study by Tukey et al. [36] showed data of efficacy of CE, with the overall sensitivity for the diagnosis of CD of 85%, specificity of 73%, PPV of 31%, and negative predictive value of 97%, but when the test characteristics were determined according to CE findings alone, those patients with >3 small bowel ulcers had a PPV of 50% for CD and, if assessed only in patients under 30 years, the sensitivity of CE is 100%, specificity 78%, and PPV 67% and in assessed selected group (ICCE's criteria about suspected CD) with a pretest likelihood of 50% CE led to a posttest probability of 85%. CE has a number needed to test (NNT) of 3 to yield one additional diagnosis of CD over small bowel barium radiography and NNT of 7 over colonoscopy with ileoscopy [37]. In conclusion, in patients with suspected CD, CE has a high sensitivity and negative predictive value but low PPV, although selecting patients with symptoms in addition to other objective findings may enhance the PPV of CE.

## 3. Special Situations

### 3.1. Pediatric Crohn's Disease

Unlike in adults, suspected small bowel CD is the main indication for CE in the pediatric age group [38]. If clinical indications are age-stratified, in children of age 1.5–8 years (in whom CD is less prevalent), the most common indication for CE was obscure gastrointestinal bleeding [39]. In contrast, obscure gastrointestinal bleeding in older children (older than 10 years) accounted for only 13–24% of all indications and 40–86% of CD indications [40]. In any case, it is important to know that patients with childhood-onset CD exhibit a more active disease and require more immunosuppressive and biological therapy without relation to the disease location, suggesting an intrinsic more severe phenotype [41]. Consequently, a rapid diagnosis must be made in order to treat correctly these patients.

There are fewer studies evaluating the role of capsule endoscopy in children with suspected Crohn's disease. Guilhon de Araujo Sant'Anna et al. [42] studied 20 children with suspected Crohn's disease who had negative small bowel series and colonoscopy. In 10 of them CE demonstrated multiple erosions and ulcers consistent with CD. We also used CE in 12 patients in the pediatric age [43]. The indication was a clinical suspicion of CD with a normal gastroscopy, colonoscopy, and small bowel follow-through series. Also ileoscopy was performed in 50% of the patients and no lesions were observed. In our study, CE identified lesions suggestive of CD in 7 of the 12 patients (58.3%) with the majority of the lesions located in the ileum. Similar to adults all these findings result in a change in medical therapy for 75%–92% of known CD patients in various studies [44–46]. Also CE in pediatric age can lead to reclassification of IBD from UC/IC to CD and previously diagnosed CD patients may have a more significant burden of small bowel disease. In a recent study [47] CE was used in eighteen patients, 4 previously diagnosed with CD, 4 with ulcerative or indeterminate colitis (UC/IC), and 10 “suspected” to have IBD. Following CE, 2 of 4 (50%) UC/IC patients were reclassified as having small bowel CD. In the 4 subjects with known CD, 2 (50%) had CE evidence of more proximal small bowel mucosal disease than previously recognized and, in the 10 subjects with “suspected” IBD, 8 (80%) had small bowel ulcerations leading to a definitive diagnosis of CD. These results also impacted medical decision-making in 13 of 18 (72.2%), leading to a change in medical management in 14 of 18 (77.8%). These data may help to integrate CE in evaluating IBD patients, lead to more targeted medical management changes, and improve outcomes.

### 3.2. Use of CE in Recurrence of CD

Recurrence of symptoms has been predicted by the early endoscopic appearance of lesions following ileocolonic resection for Crohn's disease. In order to avoid this recurrence and start treatment, CE has been adapted to detect postoperative recurrence of small bowel CD, but results are not conclusive. In one study CE was inferior to ileocolonoscopy detecting recurrence although lesions localized proximally were seen [48]. Unlike this, in the Pons et al. study [49] CE was more effective in the evaluation of recurrence after surgery for CD. Twenty-four patients with CD with ileocolonic anastomosis were prospectively included. Recurrences were visualized with colonoscopy in 6 patients and in 5 with CE but 10 additional recurrences were visualized only with CE. Moreover, proximal involvement was detected in 13 patients. Consequently, therapeutic management was modified in 16 patients.

It is important to conclude in this item that ileocolonoscopy should not be replaced to evaluate recurrence; however CE is a suitable alternative that can detect lesions more proximally also.

## 4. Lesions Observed by CE and Evaluation Scores for CD

**Figure 2:**
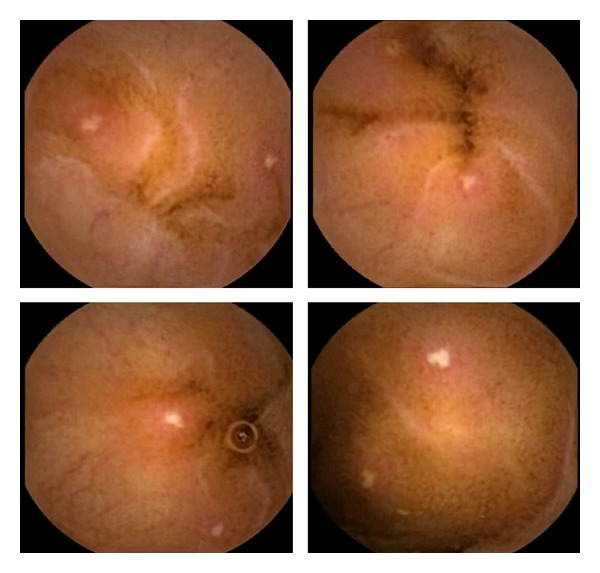
Aphthous lesions consistent with CD.

**Figure 3:**
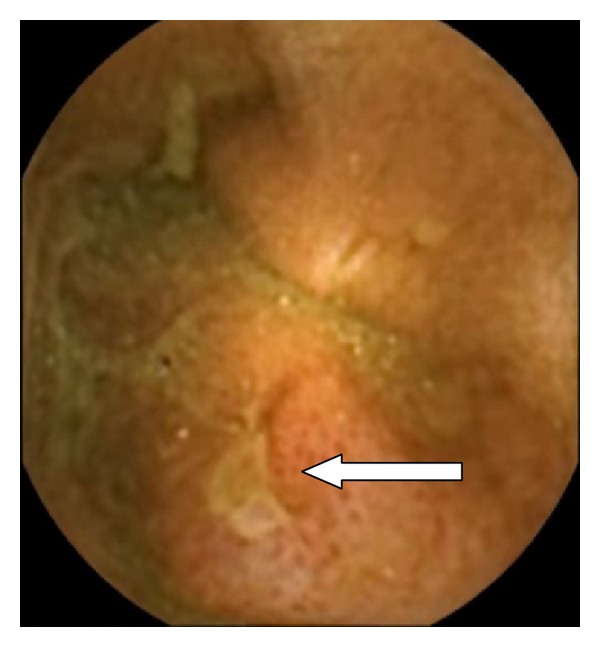
Ulcer in a user of NSAIDS.

The spectrum of the lesions observed by CE in patients with CD is varied and similar to the lesions observed by conventional endoscopy and it depends on the extent and severity of the CD. CE can usually detect mucosal fissure, linear ulcers, round ulcers, irregular ulcers, cobblestoning mucosa (composed of multiple longitudinal ulcers running parallel and hill-like elevations due to submucosal swelling), aphthous lesions, or strictured and ulcerated areas of mucosa scarring. Additionally it is able to observe bleeding lesions, polyps, and pseudopolyps suggestive of CD [50]. Other minor lesions, such as erythema, edema, loss of villi, denudated area, or aphthous ulcer, certainly not visualized by conventional radiological techniques, can be detected by CE. The main problem is that all these lesions are not specific of CD, and the differential diagnosis must be done mainly with lesions induced by NSAIDS, because some small-bowel lesions may be found in up to 75% of NSAID users, even after 2 weeks' ingestion of such drugs [51, 52] (Figures 2 and 3).

Some studies have been developed to define what type or number of lesions could be more specific of CD. Mow et al. [53] published their experience in the use of CE in patients with Inflammatory Bowel Disease. Nine patients out of 22 were given a diagnosis of definite CD (40%) based on findings of linear erosions and multiple ulcerations by capsule study. Of these 9 patients, 5 had subsequent histological findings in agreement with capsule and clinical diagnosis of CD. Outcome measures were classified as diagnostic when multiple ulcerations were present and suspicious when ≤3 ulcerations were seen. Also, some evaluation systems for inflammatory diseases of the small bowel mucosa detected by capsule endoscopy have been designed. The Lewis Index [54] has been newly developed and scores 3 parameters: villous edema, ulceration, and stenosis (which are weighted based on extent and severity). A score lower than 135 is considered normal or clinically insignificant; scores between 135 and 790 are classified as mild and scores higher than 790 as moderate to severe. Nevertheless, the score cannot specify the etiology of the mucosal inflammatory changes observed. The Lewis score has been integrated into the last software from the PillCam (Given, Rapid Reader) making it more accessible. In one recently published study 56 patients underwent CE for suspected CD using the Lewis Score and the ICCE criteria, concluding that patients not fulfilling the ICCE criteria have lower Lewis score and therefore fewer are diagnosed with CD during follow-up [55]. In this study patients were divided into three groups, according to clinical presentation: Group 1 (28 patients), suspected CD not supported by the International Conference on Capsule Endoscopy (ICCE) criteria; Group 2 (19 patients), suspected CD based on two ICCE criteria; and Group 3 (9 patients), patients fulfilling three or more criteria. Inflammatory activity was assessed with Lewis score. CE detected significant inflammatory activity (LS ≥ 135) in 23 patients (41.1%), 5 patients from Group 1 (17.8%), 11 from Group 2 (57.9%), and 7 from Group 3 (77.8%) (*P* < 0.05). CD was diagnosed in 23 patients (41.1%): 6 patients from Group 1 (21.4%), 10 from Group 2 (52.6%), and 7 from Group 3 (77.8%) (*P* < 0.05). CD was diagnosed in 82.6% of patients with significant inflammatory activity on CE (LS ≥ 135), but in only 12.1% of those having a LS < 35 (*P* < 0.05). They conclude that Lewis score has a positive predictive value of 82,6%, a negative predictive value of 87,9%, a sensitivity of 82,6%, and a specificity of 87.9%. Korman et al. [56] proposed the capsule endoscopy structured terminology (CEST), which has been adopted as the terminology to be used for lesion description. Investigators used the CEST to create a description of a given finding (erythema, edema, nodularity, ulcer, and stenosis), number of findings, distribution pattern, longitudinal extent, shape, and size to create this scoring system. Gal et al. [57] also published a similar scoring system for CE titled capsule endoscopy Crohn's disease activity index (CECDAI) which includes evaluation of 3 parameters including inflammation, extent of disease, and presence of stricture, all graded on a numeric scale with the small bowel divided into proximal and distal halves. The authors reported a kappa for the final score for each patient between different evaluations that was 0.87.

Although these scoring systems can quantitatively describe the number and severity of mucosal abnormalities detected, they had no utility in distinguishing different diagnostic entities. In addition, no scoring system has been shown to have a relationship to the patient's clinical status or CDAI scores, so more studies must be done to improve or perform new score systems.

## 5. Complications

Although CE is able to observe small bowel lesions better than other image techniques, CE retention is one important limitation. The ICCE consensus statement defined capsule retention as having a capsule endoscope remaining in the digestive tract for a minimum of two weeks [18]. The risk of retention is 1.5% when CE is performed in the setting of suspected CD, but 13% of cases have been reported in studies performed in the setting of previously known CD [64]. The choice of surgical, endoscopic, or medical management once capsule retention has been diagnosed depends on the cause of the retention, the indication for the exam, and previous treatment. A retained capsule is usually asymptomatic but may be associated with symptoms of partial or complete bowel obstruction or bowel perforation that has been reported [65]. In any case, there is not a consensus on the timing of intervention and how long one should wait in asymptomatic patients.

In an attempt to avoid this complication, a dissolving test capsule called “patency capsule” [66] was developed. It allows assessed patient with probable CD to safely undergo a CE, despite clinical and radiographic evidence of small bowel stenosis in CD. The patency capsule is swallowed by the patient and the scan is 30 hours after ingestion. Thirty-eight percent of patency capsule is dissolved after 35 h and all are dissolved between 36 and 72 h. Some cases of intestinal occlusion have been reported with the patency [67], so a new capsule with two-timer plus (Agile^©^ patency capsule) has recently been developed in order to minimize the risk of occlusion. A clinical trial [68] has demonstrated that it is a useful, noninvasive tool to identify which patients with suspected strictures could safely ingest the standard video capsule. In cases of an unsuccessful patency capsule procedure, the small bowel should be investigated by radiological imaging such as MRE or by enteroscopy. It is important to enhance that normal radiographic studies cannot entirely exclude the potential for small-bowel capsule retention and in case of a stricture suspected the Agile^©^ patency capsule should be considered [24].

## 6. Conclusions

Capsule endoscopy is a good method to evaluate the small bowel resulting in better outcomes of diagnosis, classification, therapeutic management, and prognosis of patients with CD. Nevertheless there remains much to be clarified: it is necessary to improve the specificity of the CE and to determine the place of the CE in the recurrence of CD and, for example, its role in monitoring drug response.

## Figures and Tables

**Table 1:** Comparative yield of CE and cross-sectional imaging (CSI) in patients with suspected CD (modified from Doherty et al. [58]).

Study	CSI technique	Patients	Yield of CE	Yield of CSI
Voderholzer et al. [59]	CT enteroclysis	*N* = 41	61%	29%

Eliakim et al. [16]	CT enteroclysis	*N* = 35	77%	50%

Hara et al. [60]	CT enteroclysis	*N* = 17	71%	53%

Jensen et al. [21]	CT enteroclysis MRE	*N* = 93	30% Sens.: 100% Spec.: 91%	**CTE: 33%** Sens.: 76% Spec.: 85% **MRE: 28%** Sens.: 81% Spec.: 86%

Casciani et al. [61]	MRE	*N* = 60	Sens: 90,9% Spec: 92,3%	Sens.: 100% Spec.: 97,6%

Tillack et al. [62]	MRE	*N* = 19	95%	95%

Albert et al. [63]	MRE	*N* = 52	93% Sens.: 92% Spec: 100%	88% Sens.: 77% Spec: 80%
